# Fasudil Loaded PLGA Microspheres as Potential Intravitreal Depot Formulation for Glaucoma Therapy

**DOI:** 10.3390/pharmaceutics12080706

**Published:** 2020-07-27

**Authors:** Raphael Mietzner, Christian Kade, Franziska Froemel, Diana Pauly, W. Daniel Stamer, Andreas Ohlmann, Joachim Wegener, Rudolf Fuchshofer, Miriam Breunig

**Affiliations:** 1 Department of Pharmaceutical Technology, University of Regensburg, Universitaetsstrasse 31, 93040 Regensburg, Germany; raphael.mietzner@chemie.uni-regensburg.de; 2 Institute of Analytical Chemistry, Chemo- and Biosensors, University of Regensburg, Universitaetsstrasse 31, 93040 Regensburg, Germany; christian.kade@ur.de (C.K.); joachim.wegener@ur.de (J.W.); 3 Department of Human Anatomy and Embryology, University of Regensburg, Universitaetsstrasse 31, 93040 Regensburg, Germany; franziska.froemel@vkl.uni-regensburg.de (F.F.); rudolf.fuchshofer@vkl.uni-regensburg.de (R.F.); 4 Experimental Ophthalmology, University Hospital Regensburg, Franz Josef Strauss Allee 11, 93053 Regensburg, Germany; diana.pauly@ukr.de; 5 Department of Ophthalmology, Duke University, Durham, NC 27710, USA; william.stamer@dm.duke.edu; 6 Department of Ophthalmology, Ludwig-Maximilians-University Munich, Mathildenstrasse 8, 80336 Munich, Germany; andreas.ohlmann@med.uni-muenchen.de; 7 Fraunhofer Research Institution for Microsystems and Solid State Technologies EMFT, Universitaetsstrasse 31, 93040 Regensburg, Germany

**Keywords:** drug delivery, glaucoma, ROCK inhibitor, fasudil, PLGA microspheres, intravitreal injection, trabecular meshwork, Schlemm’s canal, retinal pigment epithelium, Electric Cell-Substrate Impedance Sensing

## Abstract

Rho-associated protein kinase (ROCK) inhibitors allow for causative glaucoma therapy. Unfortunately, topically applied ROCK inhibitors suffer from high incidence of hyperemia and low intraocular bioavailability. Therefore, we propose the use of poly (lactide-co-glycolide) (PLGA) microspheres as a depot formulation for intravitreal injection to supply outflow tissues with the ROCK inhibitor fasudil over a prolonged time. Fasudil-loaded microspheres were prepared by double emulsion solvent evaporation technique. The chemical integrity of released fasudil was confirmed by mass spectrometry. The biological activity was measured in cell-based assays using trabecular meshwork cells (TM cells), Schlemm’s canal cells (SC cells), fibroblasts and adult retinal pigment epithelium cells (ARPE-19). Cellular response to fasudil after its diffusion through vitreous humor was investigated by electric cell-substrate impedance sensing. Microspheres ranged in size from 3 to 67 µm. The release of fasudil from microspheres was controllable and sustained for up to 45 days. Released fasudil reduced actin stress fibers in TM cells, SC cells and fibroblasts. Decreased collagen gel contraction provoked by fasudil was detected in TM cells (~2.4-fold), SC cells (~1.4-fold) and fibroblasts (~1.3-fold). In addition, fasudil readily diffused through vitreous humor reaching its target compartment and eliciting effects on TM cells. No negative effects on ARPE-19 cells were observed. Since fasudil readily diffuses through the vitreous humor, we suggest that an intravitreal drug depot of ROCK inhibitors could significantly improve current glaucoma therapy particularly for patients with comorbid retinal diseases.

## 1. Introduction

With the advent of rho-associated protein kinase (ROCK) inhibitors netarsudil (Rhopressa^TM^) and ripasudil (Glanatec^TM^), a promising new class of drugs has been introduced for glaucoma management [1,2]. In contrast to standard treatment with prostaglandin analogs or β-blockers that reduce the intraocular pressure (IOP) but fail to tackle the root cause of IOP elevation, ROCK inhibitors target cells in the conventional outflow pathway [3]. They increase the outflow facility by acting on cells of the trabecular meshwork (TM) and Schlemm’s canal (SC). Mechanistically, they reduce stress fiber and focal adhesion formation, actomyosin contractility and the expression of various extracellular matrix (ECM) proteins. ROCK inhibitors are applied as eye drops and are therefore associated with high incidence of conjunctival hyperemia and possibly subconjunctival hemorrhage [3]. Moreover, short corneal residence time and poor corneal penetration strongly limit the bioavailability of topically applied drugs in the aqueous humor to a typical range of about 0.1% to 5% [4,5,6]. In addition, an application frequency of up to two times a day is associated with poor compliance; thus about 20% of patients discontinue therapy with eye drops after three years [4,7,8].

A delivery system that provides ROCK inhibitors to the cells of the TM and SC in a controlled and continuous fashion would significantly increase therapeutic success. To date, only a small number of sustained delivery devices have been developed, and these have been limited to traditional glaucoma drugs [9]. For example, a silicone ring placed on the cornea into the conjunctival fornix releases bimatoprost over a period of six months [9]. Reliable placement and stable localization of the ring over six months may be an issue, and the corneal barrier still poses a major hurdle for drug absorption. Recently, a bimatoprost-containing biodegradable implant for intracameral application received first approval in the USA [10]. This device tremendously increases bioavailability at the target site [11]. However, besides the risk of decellularization of corneal endothelia [10], such a freely movable device may bear the risk migrating into the posterior segment. Other preclinical approaches aim to place a drug depot subconjunctivally or supra-choroidally, but several tissue barriers severely reduce the amount of drug that reaches its place of action, and rapid drug elimination into the circulation increases off-target effects [12].

**Scheme 1:**
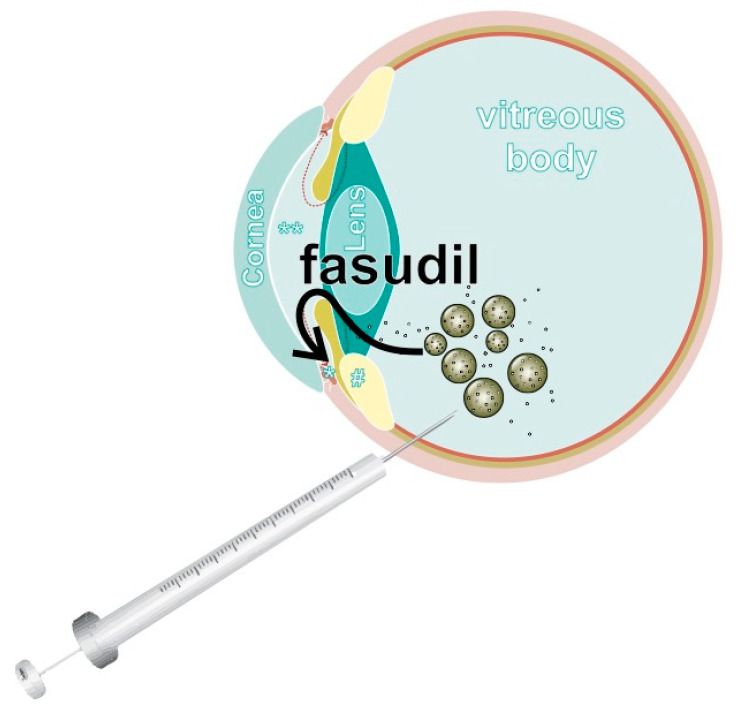
Fasudil-loaded microspheres injected into the vitreous body. After intravitreal injection, microspheres agglomerate and subsequently sediment at the site of injection leaving a free visual axis [23]. As the depot formulation degrades, fasudil (small dots) is released and transported to its target site in the anterior chamber (**) of the eye, taking advantage of natural water movement patterns in the eye. Red dashed arrow indicates the flow of the aqueous humor. The dimensions do not correspond to reality. #: ciliary body; *: Trabecular meshwork and Schlemm’s canal. With permission adapted from Mietzner et al. [4].

We propose to create an intravitreal depot of ROCK inhibitors because the vitreous offers space for a huge drug reservoir of about 100 µL and intravitreal injections have become routine in the clinic [6,13,14]. Intravitreal depot formulations have gained significant attention for the delivery of antibodies or small molecules to the posterior eye [15,16,17], however, they have not been exploited to deliver drug to the cells in the anterior chamber. This transport route is feasible since small molecules are eliminated from the vitreous via both the anterior chamber and the retina [13,18]. We hypothesize that the ROCK inhibitor will be transported to the anterior chamber after release from such an intravitreal depot (Scheme 1). The great advantages of this strategy are that there is no ocular surface exposure and the portion of the drug that reaches the retina may be beneficial. In fact, ROCK inhibitors have been demonstrated to have neuroprotective effects on cells of the retina [19].

In this study, poly (lactic-co-glycolic acid) (PLGA) microspheres containing the ROCK inhibitor fasudil were developed. We selected PLGA as the material for the depot formulation because it is a biodegradable polymer and because PLGA microspheres are well tolerated in the rat vitreous up to a concentration of two milligrams per milliliter [20]. Depending on microsphere size, polymer composition and concentration, PLGA microspheres allow for releasing small molecules up to 90 days [20,21] which would be a convenient dosing interval for glaucoma treatment. We demonstrated the principal feasibility of our approach by diffusion experiments of fasudil through porcine vitreous ex vivo as well as measurements of the functionality of TM and SC cells that received fasudil released from microspheres. Fibroblasts were included as well because during glaucoma development, cells of the TM experience a change from a mesenchymal- to myofibroblast-like phenotype [22]. In addition, a potential adverse effect of fasudil on adult retinal pigment epithelium cells (ARPE 19 cells) was investigated.

## 2. Materials and Methods

### 2.1. Materials

Ester-terminated PLGA (ratio 50:50; Resomer^®^ RG 503) was acquired from Evonik (Darmstadt, Germany). Fasudil HCl was supplied by Selleck chemicals (Houston, TX, USA). Dichloromethane (DCM) was purchased from Sigma-Aldrich (Taufkirchen, Germany). Ultrapure water was obtained from a Millipore system (Millipore, Schwalbach, Germany). Polyvinyl alcohol (PVA; Mowiol 8–88) was obtained from Kuraray Specialities Europe GmbH (Hattersheim am Main, Germany). Tween-20 was purchased from SERVA Electrophoresis GmbH (Heidelberg, Germany). Dulbecco’s modified Eagle’s medium (DMEM) for cell culture was purchased from Pan Biotech GmbH (Aidenbach, Germany).

### 2.2. Preparation of Fasudil-Loaded PLGA Microspheres

PLGA microspheres were prepared by double emulsion solvent evaporation technique. Two modifications of the water-in-oil-in-water (W/O/W) and one solid-in-oil-in-water (S/O/W) emulsification manufacturing method were applied. The main characteristics of the methods are summarized in Table 1: S/O/W microspheres (S) were prepared according to Xu et al. with slight modifications [24]. First, fasudil was crushed with a mortar and micronized by two cycles of jet milling (MC One^®^, Jetpharma SA, Balerna, Switzerland) at 6 bar and room temperature (microsphere size ≈ 1 µm). Micronized fasudil HCl (20 mg) was added to 1 mL DCM containing 200 mg PLGA and dispersed for one minute by probe ultrasonication (Digital Sonifier Model 250-D Branson, MO, USA) in an ice–water bath. Then, 10 mL of 1% (*w/v*) PVA solution were added to the primary dispersion and homogenized (7000 rotations per minute (rpm); 30 s) at room temperature using a T18 digital Ultra-Turrax equipped with an S 18N–10G dispersing tool (IKA Labortechnik, Staufen, Germany). The resulting S/O/W multi-emulsion was rapidly poured into 90 mL of 1% (*w/v*) PVA solution and stirred for three hours using a magnetic stirrer (700 rpm). The resulting microspheres were collected by centrifugation (3000 rpm) and washed three times with ultrapure water. The PLGA microspheres were finally lyophilized with an LMC-2 freeze-dryer (Christ, Osterode am Harz, Germany) and stored at −20 °C until use.

The first variant of the W/O/W microspheres (W1) was prepared according to Ramazani et al. with some modifications [25]. First 1 mL of inner water phase (ultrapure water) containing 20 mg fasudil HCl was added to 8 mL DCM containing 200 mg PLGA (25 mg/mL) and homogenized in an icewater bath at 20,000 rpm for 1 min using the same Ultra-Turrax as previously described. Next, 15 mL of 250-mM Tris HCl buffer (pH 9.0) containing 1% (*w/v*) PVA was added to the initial W/O emulsion and homogenized again for 1 min using the same homogenizer and conditions as before. The resulting W/O/W emulsion was poured into 85 mL of the same buffer (pH 9.0) containing 1% (*w/v*) PVA and stirred for 3 h using a magnetic stirrer at 700 rpm to allow the solvent to evaporate. Afterwards, the procedure was the same as for S microspheres.

The second variant of the W/O/W microspheres (W2) was prepared by dissolving fasudil HCl (5 mg) in 500 µL of ultrapure water. Then, 3 mL of DCM containing 200 mg PLGA (67 mg/mL) was added to the initial fasudil solution and homogenized at 10,000 rpm for 1 min using the Ultra-Turrax. This primary W/O emulsion was subsequently added to 10 mL of 1% (*w/v*) PVA solution and homogenized again at 10,000 rpm for 1 min. The resulting W/O/W emulsion was poured into 90 mL of 1% (*w/v*) PVA solution and stirred again for 3 h using a magnetic stirrer at 700 rpm, allowing the solvent to evaporate. Afterwards, the procedure was the same as for the other two samples (W1 and S). Three independent batches of each microsphere type were produced.

### 2.3. Characterization of Fasudil-Loaded PLGA Microspheres

#### 2.3.1. Size Determination

The volume weighted mean diameter (reported as microsphere size in the following) and microsphere size distribution of the microspheres were determined by laser diffraction spectroscopy after the washing step in the manufacturing procedure. Analyses were performed with a Mastersizer 2000 (Malvern Panalytical Ltd., Malvern, UK) equipped with a Hoydro 2000 µ dispersion unit. For the measurement, microspheres were dispersed in ultrapure water (≈8 mg/mL) and were added into the dispersion unit, which was floated with ultrapure water, until the obscuration was higher than 5%. The stirring speed was set to 500 rpm. To determine the uniformity of the microsphere sizes, span values were calculated according to the following equation:

(1) $$Span=\left(\frac{d90-d10}{d50}\right)$$

d90, d10 and d50 are the microsphere diameters at 90%, 10% and 50% of the cumulative size, respectively. Data were obtained by three independent samples, presented as mean ± standard deviation.

#### 2.3.2. Morphologic Characterization

The surface morphology of fasudil-loaded microspheres was observed with a Zeiss LEO 1530 Gemini scanning electron microscope (Carl Zeiss Microscopy GmbH, Oberkochen, Germany). Lyophilized microspheres were placed to conductive pads (Plano GmbH, Wetzlar, Germany) stuck to aluminum specimen stubs. The samples were sputtered with gold two times for two minutes using a Polaron E5100 coating unit (Polaron Equipment, Ltd., Hertfordshire, UK).

#### 2.3.3. Fasudil Quantification

Fasudil was quantified by high performance liquid chromatography (HPLC) using a 1260 Infinity II LC system (Agilent Technologies, Inc., Santa Clara, CA, USA) equipped with a binary pump and a diode array detector. Analyses were performed using a reverse phase C_18_ column (150 mm × 5 mm; 5-µm microsphere size; Eclipse XDB-C18, Agilent Technologies, Inc., Santa Clara, CA, USA) preceded by a C_18_ security guard cartridge (Phenomenex, Inc., Torrance, CA, USA) at 40 °C. The mobile phase was composed of ultrapure water (eluent A) and methanol (eluent B; HPLC grade, Merck, Darmstadt, Germany), both containing 0.03% (*v/v*) trifluoroacetic acid (HPLC grade, Sigma-Aldrich GmbH, Taufkirchen, Germany). Fasudil was eluted by a gradient at a flow rate of 0.8 mL/min. The gradient used was as follows: 0.0–0.5 min constant at 85% (*v/v*) eluent A; 0.5–6.5 min 85–40% (*v/v*) eluent A (linear gradient); 6.5–7.0 min changed to 5% (*v/v*) eluent A; 7.0–17.0 min constant at 5% (*v/v*) eluent A; 17.0–17.10 min changed to 85% (*v/v*) eluent A; 17.10–27.10 min constant at 85% (*v/v*) eluent A. The injection volume was 5 µL for encapsulation efficiency (% EE) measurements and 10 to 12 µL for in vitro release studies. Each sample was injected three times. The absorbance of fasudil was measured at λ = 320 nm.

#### 2.3.4. Encapsulation Efficiency

Fasudil content of microspheres was quantified by dissolving lyophilized fasudil-loaded microspheres (20–40 mg) in DCM (1 mL), followed by precipitation of PLGA by the addition of methanol (3 mL). The obtained dispersion was mixed, centrifuged (9000 G; 10 min), and the supernatant was filtered for fasudil quantification by HPLC-UV-Vis. To calculate the percentage encapsulation efficiency (% EE) and drug loading (% DL), the following equations were used:

(2) $$\%\;EE=\left(\frac{actual\;drug\;content}{theoretical\;drug\;content}\right)\times 100$$

(3) $$\%\;DL=\left[\frac{drug\;\left(mg\right)}{drug+polymer}\right]\times 100$$

Three independent samples were analyzed and are presented as mean ± standard deviation.

#### 2.3.5. In Vitro Drug Release

Fasudil-loaded microspheres (20–50 mg) of three independent batches were dispersed in 1 mL of Dulbecco’s phosphate-buffered saline (DPBS) containing 0.02% Tween-20 in 2-mL centrifuge tubes and placed in a shaking water bath incubator (37 °C). Tween-20 was added to improve the wettability and to avoid floating of microspheres. At several points over a time period of 50 days, samples were centrifuged and 500 to 850 µL of the supernatants were replaced by the same volume of fresh buffer. The pellet was redispersed, placed back in the incubator and collected supernatants were stored at −80 °C until fasudil quantification. Data were obtained from three independent samples and are expressed as mean ± standard deviation. Because the amount of released fasudil was rather low, the drug was pooled from the release medium of all three microsphere types for subsequent experiments.

#### 2.3.6. Mass Spectrometric (MS) Analysis of Fasudil

Analyses were performed with an Agilent 1290 Infinity HPLC system (Agilent Technologies, Inc., Santa Clara, CA, USA), interfaced to an Agilent 6540 Quadrupole time-of-flight (Q-TOF) mass spectrometer with a dual electrospray ionization (ESI) source (Agilent Technologies, Inc., Santa Clara, CA, USA). A Phenomenex (Torrance, CA, USA) Luna Omega 1.6 µm C18 column (pore size 100 Å; 100 × 2.1 mm) was used for separation at 40 °C. The mobile phase was composed of ultrapure water (eluent A) and acetonitrile (eluent B), both containing 0.1% (*v/v*) formic acid. The elution was performed under gradient conditions at a flow rate of 0.6 mL/min. The flow gradient used was as follows: 0–4 min 95–2% (*v/v*) eluent A (linear gradient); 4–5 min constant at 2% (*v/v*) eluent A; 5–5.10 min changed to 95% (*v/v*) eluent A. Mass spectrometric analyses were carried out in positive ion mode.

#### 2.3.7. Preparation of Released Fasudil for Cell Experiments

To investigate the biologic activity of fasudil released from microspheres (released fasudil), parallel to in vitro release studies, fasudil-loaded microspheres were incubated in ultrapure water containing 0.02% Tween-20 for several days. The supernatant was collected and lyophilized. The lyophilized product was reconstituted in ultrapure water, and after adjusting the pH to 7.3, the solution was sterile filtered, and the amount of fasudil was determined by the previously described method. Finally, the solution was diluted to the desired concentration with DPBS. The same procedure was followed for control microspheres (unloaded microspheres) except for fasudil quantification.

### 2.4. Cellular Effect of Fasudil

#### 2.4.1. Cell Culture

Primary human trabecular meshwork cells (TM cells) and Schlemm’s canal cells (SC cells) were used until passage numbers 7 and 12, respectively. Primary cultures of human foreskin fibroblasts were used until passage 13. TM and SC cells were characterized according to established methods [26,27]. Procedures for securing human tissue were humane, included proper consent and approval accordingly to the Declaration of Helsinki. Primary cells were cultivated in DMEM (supplemented with 4.5 g/L glucose for fibroblasts and 1 g/L glucose for TM and SC cells) containing 10% (*v/v*) fetal bovine serum (FBS; Thermo Fisher Scientific, Waltham, MA, USA), 100 units/mL penicillin and 100 µg/mL streptomycin. Immortalized human TM cells (HTM-N) were used for electric cell–substrate impedance sensing (ECIS) and were made available by Iok-Hou Pang and Louis DeSantis (Alcon Research Laboratories, Fort Worth, TX, USA). They were cultured according to published protocols [28]. Human male adult retinal pigment epithelium cells (ARPE-19 cells, American Type Culture Collection, #CRL-2302 passage 25) were cultivated in Transwell^®^ inserts for 4–6 weeks in DMEM containing 4.5 g/L glucose supplemented with 1% FCS (Pan Biotech GmbH, Aidenbach, Germany), 100 units/mL penicillin, 100 µg/mL streptomycin and 1 mM sodium pyruvate. If not otherwise stated, cells were incubated for 24 h in medium containing 0.35% FBS supplemented with unencapsulated fasudil (free fasudil; 25 µM) or released fasudil (25 µM), respectively. Both, DPBS and medium collected from blank microspheres served as negative controls for free and released fasudil, respectively. Since there was no significant difference between both controls, only one representative control is shown. A serum concentration of 0.35% was chosen because it is equivalent to the amount of protein in the aqueous humor [29]. Fasudil at a concentration of 25 µM was used because it is a widely applied concentration and was well tolerated by the cells (c.f. supplementary materials: Figure S1) [30,31]. Cells were not directly incubated with the microspheres, but rather with the release media containing fasudil. Since the loading with fasudil was too low, an immensely high number of microspheres would be necessary to elicit cellular effects. In this case, a strong acidification of the culture medium would have occurred due to PLGA’s degradation products glycolic and lactic acid.

#### 2.4.2. Fluorescence Labeling of Actin Cytoskeleton

Cells were placed into 8-well μ-slides (ibidi GmbH, Gräfelfing, Germany) at a density of 2 × 10^4^ cells/well. After fasudil exposure, as described above, cells were washed once with 0.1×-PBS, fixed in 4% paraformaldehyde for 5 min at room temperature and washed twice with 0.1×-PBS for 5 min. To stain actin stress fibers, fixed cells were incubated in the dark with phalloidin–fluorescein isothiocyanate (FITC–phalloidin; Invitrogen, Molecular Probes, Eugene, OR, USA) for 1 h at room temperature. Afterwards, cells were rinsed three times with 0.1×-PBS and embedded in Dako Fluorescence Mounting Medium (Agilent Technologies, Santa Clara, CA, USA). Nuclei of ARPE19 cells were labeled additionally by 4′,6-diamidino-2-phenylindole (DAPI). The fluorescence of fibroblast, TM and SC cells was visualized using an LSM 510 Meta confocal microscope (Carl Zeiss AG, Göttingen, Germany)). To visualize ARPE19 cells, an Axio Imager Z1 fluorescence microscope (Carl Zeiss AG, Göttingen, Germany) was used.

#### 2.4.3. Collagen Gel Contraction Assay

The collagen gel contraction assay was carried out with slight modifications according to Su et al. [32]: Fibroblasts, TM cells, and SC cells were trypsinized (Pan Biotech GmbH, Aidenbach, Germany) and resuspended at a density of 1.5 × 10^5^ cells/mL in DMEM containing 0.35% FBS and 1.17 mg/mL of rat tail collagen type I (4.00 mg/mL in 0.02-N acetic acid; BD Bioscience, San Jose, CA, USA; (adjusted with 0.02-N acetic acid for the desired concentration). To allow the gel to solidify at optimal conditions, 1-M sodium hydroxide solution was added to adjust the solution to neutral pH. Then, 500 µL of the collagen-cell mixture were transferred to each well of a 24-well plate. The mixture was incubated at 37 °C under 5% CO_2_ for one hour. After the gel solidified, 500 µL of DMEM containing 0.35% FBS supplemented with controls, free or released fasudil for a final concentration of 25 µM was pipetted onto the collagen gels. Gels were carefully dissociated from the wall of the culture wells using a hypodermic needle. After 0, 24, 48 and 72 h, culture wells were tangentially illuminated and photographed (EOS 750D, Canon, Tokyo, Japan) from above with a fixed distance in front of a dark background. The areas of the matrices were measured using ImageJ software (version 1.52d, U.S. National Institutes of Health, Bethesda, MD, USA) [33]. For normalization, the mean areas measured in controls was set at 1 (*n* = 4).

#### 2.4.4. Electric Cell–Substrate Impedance Sensing (ECIS)

The impact of fasudil on HTM-N cells was monitored by noninvasive impedance readings. A schematic illustration of the experimental setup is shown in Section 3.5. HTM-N cells were seeded in 8-well plates milled into a poly(methyl methacrylate) block with a well diameter of 16 mm each. In order to allow for ECIS measurements, the bottom of the well was made from a polycarbonate (Lexan^®^) base substrate coated with a gold electrode layout generated by sputter deposition of gold and subsequent photolithographic patterning. The bottom plate with the electrode layout was glued to the 8-well block using a biocompatible silicon adhesive. Cells were either treated directly by replacing half of culture medium with fresh culture medium containing controls, free or released fasudil for a final concentration of 25 µM; or samples were premixed with 200 µL vitreous body of fresh enucleated porcine eyes (purchased from a local slaughterhouse) by vortexing them and putting them into 6.5 mm Transwells^®^ with a polycarbonate membrane holding 108 pores per cm² of 0.4 µm pore diameter each (Corning, Inc., Corning, NY, USA). The height of the vitreous body in the insert was 6 mm. Experiments were performed at 37 °C under 5% CO_2_. Relay bank, lock-in amplifier and software for the ECIS data acquisition and analysis were obtained from Applied BioPhysics (Troy, NY, USA). The impedance values of each well were recorded at an alternating current frequency of 8 kHz every 4 min over the entire time of analysis. Impedance values are presented as the values captured along the experimental time course normalized to the impedance values recorded immediately before addition of test substances. Two independent experiments were performed. Due to the 8-well-format of the experimental setup, all fasudil containing samples (free- and released fasudil) were performed in triplicate and control samples were performed in duplicate.

### 2.5. Statistical Analysis

All data are presented as means ± standard deviation. Standard deviations of normalized mean values were calculated according to the rules of error propagation. Multiple *t*-tests with the Holm–Sidak method (in Section 3) were performed using GraphPad Prism 6.0c (GraphPad Software, San Diego, CA, USA) to assess statistical significance (statistical significance assigned at *p* < 0.05).

## 3. Results

### 3.1. Manufacture and Characterization of Fasudil-Loaded Microspheres

**Figure 1:**
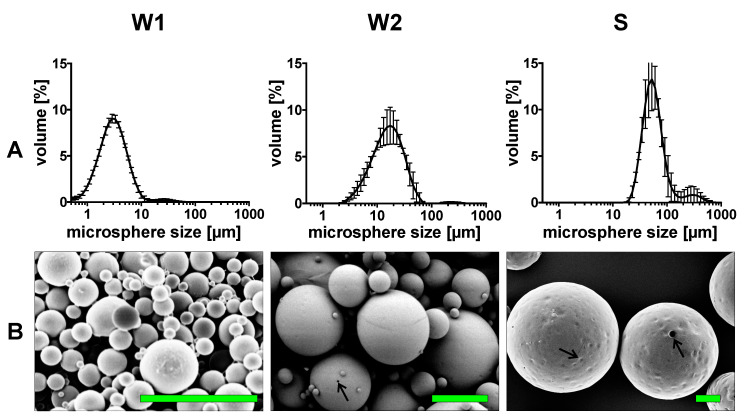
Microsphere size distribution and shape of microspheres. (**A**) Volume microsphere size distribution of W1, W2 and S microspheres was measured by laser light diffraction. The W1 batch had the smallest microsphere size of about 3.4 µm, followed by W2 (18.2 µm) and S with a microsphere size of about 66.9 µm. Error bars represent the standard deviation (SD) of the volume microsphere size of three independent batches; (**B**) Shape of the microspheres was visualized by scanning electron microscopy (SEM). All microsphere types had a round shape. W1 microspheres were quite smooth and nonporous. In contrast, some W2 and S microspheres exhibited holes (white arrows) on their surface, and S microspheres additionally showed circular dents as indicated by the blue arrow. Bars indicate 10 µm.

Because fasudil is a water-soluble drug, the double emulsion solvent evaporation technique was chosen for its encapsulation into PLGA microspheres. Two modifications of the water-in-oil-in-water (W/O/W) and one solid-in-oil-in-water (S/O/W) emulsification methods were applied to obtain three different microsphere species with individual sizes. These will be denoted as W1, W2 and S in the following. The W1 and W2 method differed in polymer concentration (W1: 25 mg/mL; W2; 67 mg/mL), emulsifying stirring speed (W1: 20,000 rpm; W2: 10,000 rpm) and pH of the external aqueous phase (W1: adjusted to pH 9.0; W2: not controlled). In the S preparation, the polymer concentration was 200 mg/mL and solid fasudil was incorporated into the PLGA matrix instead of dissolved fasudil, since fasudil was not soluble in DCM due to its hydrophilic nature. The W1 batch had the smallest size of about 3.4 µm, followed by W2—with a size of about 18.2 µm and S with a size of about 66.9 µm (Figure 1 and Table 2). All three microsphere types were spherical in shape as evaluated by scanning electron microscopy (SEM) (Figure 1). W1 and W2 microspheres had overall smooth and nonporous surfaces. Single W2 and S microspheres showed tiny holes on their surfaces. The surfaces of S microspheres were covered in circular dents. The encapsulation efficacy of all three batches was below 5% (Table 1). SEM images demonstrated that no free, unencapsulated fasudil was resting on the surface of microspheres.

### 3.2. Fasudil Release from Microspheres Can Be Tailored under Maintenance of its Structure

**Figure 2:**
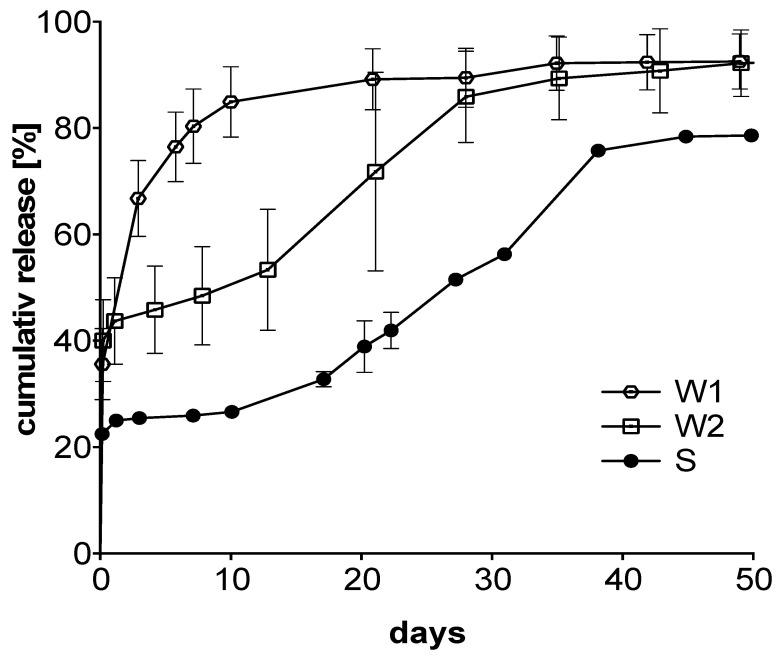
Cumulative release of fasudil from poly (lactide-*co*-glycolide) (PLGA) microspheres. Release experiments were performed in Dulbecco’s phosphate-buffered saline (DPBS) containing 0.02% Tween-20 at 37 °C. Increasing microsphere size is indicated by the gray triangle with the inscription “size.” The burst release of fasudil was reduced with increasing microsphere size. In the same order, the release period was extended. Data are expressed as mean ± standard deviation of cumulative released fasudil of three independent batches.

The release of fasudil depended strongly on the microsphere size of the microspheres (Figure 2). The smallest microspheres (W1) had a high burst release of about 65% during the first 3 days, and the monophasic release was completed after only 20 days. Medium-sized W2 microspheres also showed a high burst release of about 40%, which was followed by a sustained release period over 35 days. In contrast, the S microspheres showed a typical triphasic sustained release, over about 45 days starting with a burst release of only about 20% followed by a plateau from day 1 to day 10 where no fasudil was released up to day 10 and followed by an almost linear release from day 17 to day 38.

**Figure 3:**
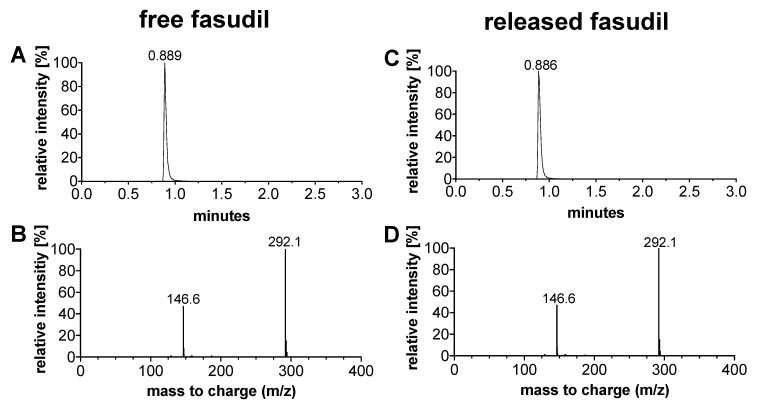
Integrity of fasudil before encapsulation and after release from PLGA microspheres. (**A**, **B**) HPLC-MS total ion chromatogram and electrospray mass spectrum of free fasudil. (**C**, **D**) HPLC-MS total ion chromatogram and electrospray mass spectrum of released fasudil. Both, free and released fasudil had a retention time of 0.89 min and the molecular peak was at 292.1 *m/z*.

To determine whether the fasudil was chemically unmodified after release from microspheres, HPLC-mass spectrometry (HPLC-MS) analysis was performed. Figure 3a shows the HPLC-MS total ion chromatogram of fasudil that was not encapsulated into microspheres as reference (indicated as free fasudil), and Figure 3b shows fasudil after it was released from microspheres (indicated as released fasudil). Both, free and released fasudil had the same retention time of 0.89 min. The MS-spectra of free and released fasudil matched very well (Figure 3c,d). Both showed a molecular peak at 291.1 *m/z* and a fragment ion peak at 146.6 *m/z* indicating that fasudil remained structurally intact during the encapsulation process and release.

### 3.3. Released Fasudil Reduces Actin Stress Fiber Formation

**Figure 4:**
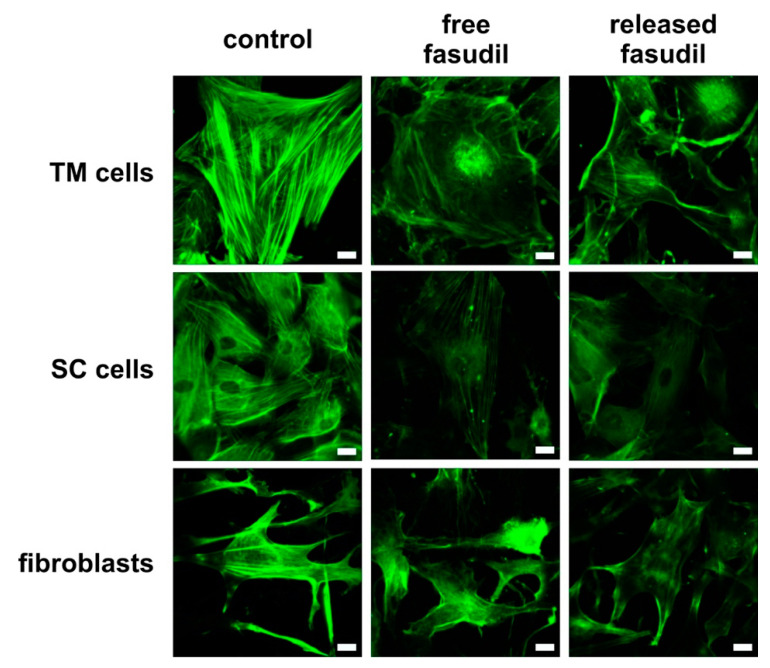
Free and released fasudil disrupt actin stress fibers. Representative fluorescence microscopic images of the actin cytoskeleton. Human using trabecular meshwork (TM) and Schlemm’s canal (SC) cells and fibroblasts were incubated for 24 h with free or released fasudil at a concentration of 25 µM. This concentration was chosen because it is widely used in literature and well tolerated by all three cell types at a serum concentration of 0.35% (c.f. supplementary materials Figure S1) [30,31]. Actin cytoskeleton was labeled with FITC–phalloidin and visualized by fluorescence microscopy. Free and released fasudil caused a reduction of actin stress fibers and induced an actin reorientation compared to controls. Scale bar indicates 20 µm.

ROCK inhibitors decrease the outflow resistance in the anterior chamber of the eye due to their effects on the cytoskeleton of TM and SC cells [4,34]. TM and SC cells as well as fibroblasts were treated with free fasudil and fasudil released from microspheres. After staining the actin cytoskeleton with FITC–phalloidin, cells were studied by fluorescence microscopy (Figure 4). Untreated TM and SC cells and fibroblasts displayed numerous thick and longitudinally arranged actin stress fibers. The stress fibers of TM cells and fibroblasts were thicker than those of SC cells, which had a more cortical actin at baseline. In contrast, fasudil treatment (free or released) caused a dramatic reduction of actin stress fibers, and rather a cortical actin cytoskeleton remained. In all cell types, the effect of fasudil released from microspheres was comparable to free fasudil, corroborating that fasudil was not affected by the encapsulation procedure and still biologically active after release.

### 3.4. Released Fasudil Reduces Cell Contractility

**Figure 5:**
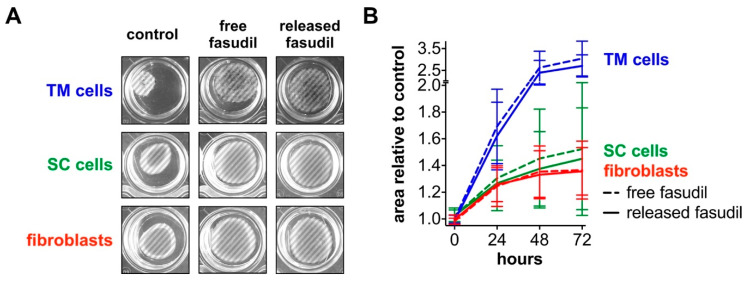
Free and released fasudil reduce contractility of fibroblasts, TM and SC cells embedded in collagen gels. (**A**) Representative images of control gels and gels that were incubated with free or released fasudil at a concentration of 25 µM after 48 h. The projected gel surface is highlighted by the hatched area. Control gels with embedded TM cells had the smallest surface area. The surface areas of gels with SC cells or fibroblast were similar. After incubation with fasudil, the surface area was much larger irrespective of the cell type. (**B**) Semiquantitative analysis of mean areas of collagen gels incubated with free or released fasudil normalized to controls at 0, 24, 48 and 72 h. For each cell type, no significant difference (*p* < 0.05) between free or released fasudil treated gels was observed. Y-axis is discontinued between 2.0 and 2.5. Data are expressed as normalized mean ± standard deviation of the mean (*n* = 4).

To functionally measure the effect on actomyosin contractility, TM and SC cells as well as fibroblasts were embedded in a three-dimensional collagen type I gel and treated with free or released fasudil. Because cells have an inherent contractility, the gel matrix moves together over time. As a consequence, the gel surface area decreases. The more pronounced the cell contractility is, the smaller is the gel surface area and vice versa. The gel surface area was analyzed over time and served as a measure for cell contractility [35,36]. Gel area and cell contractility behave inversely, which means the smaller the gel area, the higher is the cell contractility. The gels initially filled the entire well, but over time and without fasudil treatment, the surface area of control gels decreased due to the contractility of embedded cells (Figure 5a, left column). Control gels with embedded TM cells had the smallest surface area after an assay time of 72 h, followed by those with SC cells and fibroblasts. In contrast, with fasudil treatment (free or released), the gel surface area remained nearly constant or only slightly decreased over 72 h (Figure 5a, middle and right columns). Due to the stronger gel contraction observed for TM cells under control conditions, the impact of fasudil was more pronounced for TM cells than for fibroblast and SC cells. This became even more apparent in the semi-quantitative analysis in Figure 5b. After 48 h, for example, the projected gel area relative to the control was the highest when TM cells (~2.4-fold) were embedded, followed by SC cells (~1.4-fold) and finally fibroblasts (~1.3-fold). For each cell type, no significant difference (*p* > 0.05) between free or released fasudil-treated cells was observed.

### 3.5. Fasudil Diffuses through Vitreous Humor and Alters Cell Junctions

**Figure 6:**
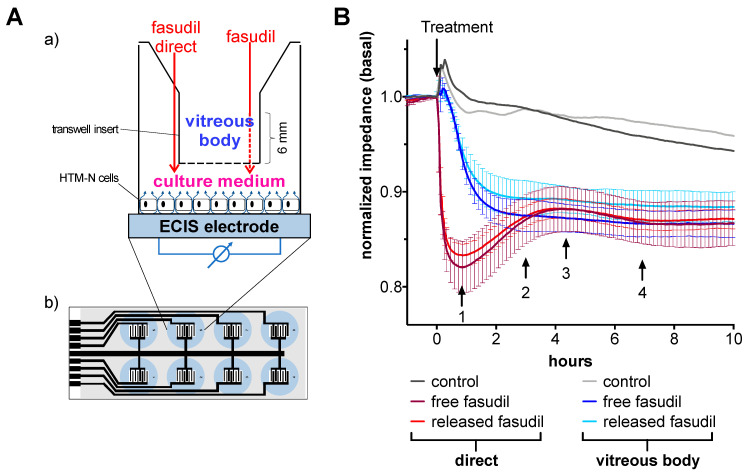
Free and released fasudil diffuse through the vitreous body and induce changes in cell–cell and cell–matrix junctions of immortalized TM cells. (**A**) Schematic illustration of the experimental setup of the vitreous diffusion assay in combination with electric cell–substrate impedance sensing (ECIS). (**a**) Cells were seeded in a culture well with integrated gold-film electrodes to allow for impedance measurements. Impedance was recorded at an alternating current frequency of 8 kHz. Cells were treated with free or released fasudil at a final concentration of 25 µM. Fasudil was either directly added to the cells (solid red arrow) or first mixed with vitreous humor, which was then placed in a Transwell^®^ insert (dashed red arrow). Small blue arrows represent the current flow. (**b**) The bottom of the well was fabricated from a polycarbonate-based substrate (gray) with an interdigitated gold electrode layout (black). Each blue cycle represents one culture well. The dimensions are not drawn to scale; (**B**) Time-dependent changes of the impedance of immortalized TM cells (HTM-N cells) are shown. Values were normalized to impedance values immediately before addition of fasudil. Incubation with fasudil started at timepoint 0 h (indicated by arrow and “Treatment”). Cells that were directly incubated with fasudil (red curves) showed an immediate reduction of the impedance from basal values. In contrast, impedance slowly decreased over an extended time period, when a diffusion of fasudil through the vitreous was necessary (blue curves). All samples treated with fasudil (free or released) were performed in triplicate and control sample was performed in duplicate. Error bars show the standard deviation of normalized ECIS data. One representative experiment of two independent ones is shown. At indicated timepoints (black arrows with numbers), no statistical differences (*p* < 0.05) between free and released fasudil were observed.

Fasudil-loaded microspheres are intended to be injected into the vitreous body. To test whether released fasudil diffuses through the vitreous body to affect target cells in the anterior chamber, an experimental setup was developed that measures the cellular response only after successful fasudil diffusion through vitreous humor (Figure 6a). In this setup, the cellular response over time is measured by electric cell–substrate impedance sensing (ECIS) and serves as a measure for changes in cell–cell and cell–matrix junctions induced by cytoskeletal rearrangements (e.g., loss of stress fibers) [37,38]. A direct incubation of immortalized TM (HTM-N) cells with either free or released fasudil resulted in an immediate drop of impedance values from basal values by about 18% within 50 min (Figure 6b). The impedance signal showed a modest recovery over the next four hours. There was no statistically significant difference (*p* < 0.05) between free and released fasudil at any timepoint. In contrast, when fasudil was mixed with vitreous body and its contact to the cell monolayer was only possible after diffusion through the gel-like vitreous, the cellular impedance decreased over a much longer time period of about three hours to final values that were comparable to those after a direct treatment with fasudil. Again, no significant differences (*p* < 0.05) were observed between free and released fasudil. The observed impedance changes indicate that TM cells experience a significant weakening of their cell–cell and/or cell–matrix adhesion in good agreement with the fasudil-induced loss of stress fibers.

### 3.6. Released Fasudil Does Not Influence Retinal Pigment Epithelial Cells

**Figure 7:**
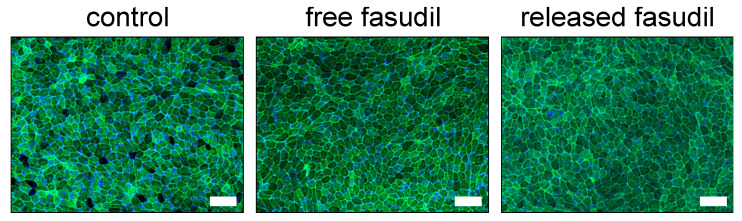
Free and released fasudil do not negatively affect retinal pigment epithelium cells (ARPE-19). ARPE-19 cells were incubated for 24 h with free or released fasudil at a concentration of 25 µM. The actin cytoskeleton was labeled with FITC–phalloidin (green) and the cell nuclei were stained with DAPI (blue). All samples had features characteristic of retinal pigment epithelium (RPE) including defined cell borders, an overall cobblestone appearance and a well-organized actin cytoskeleton. Representative fluorescence microscopic images of ARPE-19 cells are shown. Scale bar indicates 50 µm.

Fasudil that is released in the vitreous body may also come in contact with cells of the posterior chamber of the eye. Therefore, the effect of fasudil—either free or released—on the actin cytoskeleton of ARPE-19 cells was investigated. After staining the actin cytoskeleton with FITC–phalloidin, cells were visualized by fluorescence microscopy (Figure 7). Both untreated cells and cells that were treated with fasudil showed characteristics of a healthy retinal pigment epithelium (RPE), including defined cell borders, a cobblestone-like monolayer and a well-organized actin cytoskeleton, suggesting that fasudil at a concentration of 25 µM did not negatively affect ARPE-19 cells.

## 4. Discussion

ROCK inhibitors are considered as promising drug class for glaucoma therapy because they allow for causative treatment. To date, only eye drop formulations of ROCK inhibitors are available on the market. To deal with the issues associated with these formulations, including conjunctival hyperemia, subconjunctival hemorrhage and low intraocular bioavailability, we propose a depot formulation for intravitreal application. We incorporated the ROCK inhibitor fasudil into PLGA microspheres. PLGA is an advantageous material choice because it is approved by the Food and Drug Administration and the European Medicines Agency for intravitreal application [39] and it is commonly used to fabricate devices for controlled and sustained delivery of small and large drug molecules [40]. In contrast to non-biodegradable delivery devices for glaucoma therapy like the implant iDose made from titanium, which must be removed after the travoprost-loaded reservoir is empty, its biodegradability and high biocompatibility makes PLGA favorable for intraocular application.

Three modifications of the double emulsion solvent evaporation technique were applied to produce microspheres of different sizes of about 3, 18 and 70 µm (W1, W2 and S microspheres, respectively). One important parameter to control the microsphere size was the stirring speed during the first step of microsphere formation. Stirring is necessary because it provides the energy to disperse the immiscible oily PLGA phase and the aqueous drug-containing phase into each other [41]. As expected, the mean microsphere size was inversely proportional to the stirring speed. The second parameter to control microsphere size was the polymer concentration; microsphere size increased with increasing PLGA concentration. S microspheres were produced with the highest polymer concentration of 200-mg/mL and thus yielded the largest microsphere size, while W1 microspheres were the smallest and had the lowest polymer concentration (25 mg/mL) [42,43]. Size of the microspheres may have an influence on their intravitreal tolerability. Due to a higher specific surface area, smaller microspheres may show slightly more adverse effects [44]. We suggest fabrication under aseptic conditions to obtain a sterile ocular drug formulation. The encapsulation efficacy of fasudil into PLGA microspheres was only about 4%. It is known that hydrophilic molecules like fasudil with a log*P*-value around zero (log*P* = 0.16 [45]) are difficult to encapsulate into PLGA polymer matrices, which are relatively lipophilic [46]. The main reason for the poor encapsulation efficacy is that fasudil shows a free solubility in water (up to ∼200 mg/mL [47]), which favors its distribution from the lipophilic polymer matrix to the external aqueous phase. As a consequence, fasudil was already “released” from the PLGA matrix before microsphere creation was complete. Unfortunately, varying process parameters such as increasing the pH to reduce the solubility of the weakly basic fasudil (pK_a_ = 9.7 [48]) in the external water phase (as was performed for the W1 microspheres) did not improve the encapsulation efficacy [25]. In addition, the encapsulation of fasudil in its solid form, as done for the S microspheres, did not avoid its partitioning into the water phase during the manufacturing process.

Nevertheless, varying process parameters produced microspheres with different release characteristics in terms of initial release and time profile. A characteristic feature of microspheres manufactured by emulsion solvent evaporation technique is their initial burst release [49], which was particularly prominent for the smallest W1 microspheres with about 65% of the drug being released in the burst phase. This can be attributed to the high specific surface area of smaller microspheres and the shorter diffusion length that the drug must overcome to be transported from the microsphere matrix to the surrounding medium [49]. Because W2 and S microspheres were larger, the diffusion barrier to fasudil increased, resulting in initial burst releases of only about 40% and 20%, respectively. Additionally, the release duration of fasudil was prolonged to about 35 and 45 days, respectively. The PLGA concentration was the lowest for W1 (25 mg/mL) followed by W2 (66.7 mg/mL) and S (200 mg/mL) during microsphere formation. To decrease mobility and sedimentation of fasudil crystals and thus preventing drug loss towards the surrounding aqueous phase during microspheres hardening, a high polymer concentration was chosen for S microspheres to increase the viscosity. Furthermore, the polymer concentration has a huge impact on initial burst release [50]. With decreasing polymer concentration, the internal porosity is increased and vice versa [50]. A high internal porosity allows water to penetrate much easier and deeper into the polymer matrix within a shorter time frame and thus shortens the diffusion distance of the drug [50]. Additionally, hydrophilic drugs such as fasudil favor water penetration into microspheres upon drug leaching leading to higher burst release and shorter release duration [40,50]. Further possibilities to reduce the extend of burst release would be the use of a polymer with a higher molecular weight or to add additives such as alginate or chitosan to the internal aqueous phase [51,52]. S microspheres showed the typical triphasic profile, which is controlled by drug diffusion within the polymer matrix and polymer erosion [53,54]. PLGA degrades by hydrolysis into its acidic monomers, lactic and glycolic acid, and thus, the surrounding medium in which fasudil is released acidifies [55,56]; however, the integrity of fasudil was not negatively affected. Therefore, PLGA is a suitable carrier for fasudil as it does not affect its chemical structure.

Through several downstream mediators, ROCK promotes the phosphorylation of the myosin light chain and thereby facilitates the assembly and formation of actin stress fibers and focal adhesions [3,57,58]. In addition, cells of the TM undergo a switch from a mesenchymal- to a myofibroblast-like phenotype during the progression of glaucoma disease [58]. Therefore, fibroblasts were included in this study, in addition to cultures of TM and SC cells, to analyze the biologic activity of fasudil released from microspheres. Cytochemical staining clearly showed that treatment with free or released fasudil resulted in a reduction of actin stress fibers in all three cell types. Due to individual phenotypes of the three cell types and the use of primary cells from different human donors with unknown previous diseases, the number and extent of the stress fibers varied between TM and SC cells and fibroblasts. However, all three cell types showed the same trend upon fasudil treatment. Our observations were similar to effects observed in ROCK inhibitor-treated cells found in literature [31,57,59,60]. It must be kept in mind that our experimental setup measuring cellular effects of released fasudil has some limitations, since cells were only treated with a fixed dose of previously released fasudil and were not incubated directly with fasudil loaded microspheres which would reflect more the in vivo conditions.

ROCK also regulates the cellular contractility by modulating the actomyosin contraction in the outflow tissues [3,57,58]. Increased contractility of TM and particularly of SC cells alters the outflow resistance in glaucoma [61]. The impact of free and released fasudil on the cellular contractility was investigated using a collagen gel contraction assay. Treatment with fasudil caused a decrease in contraction of the collagen gel of the embedded cell type. These observations suggest reduced cellular contractility or at least altered interactions between the embedded cells and the collagen when fasudil was applied. Effects similar to those observed in our study have been reported for TM cells and fibroblasts [38,62,63]. Similar to TM cells, SC cells have a smooth muscle-like morphology and are therefore also highly contractile [61,64]. The reduced contractility of SC cells in presence of fasudil should therefore be based on a similar mechanism as for TM cells.

The present microsphere formulation is intended to be injected into the vitreous body. During PLGA degradation, fasudil most likely distributes throughout the vitreous body. Small, hydrophilic molecules like fasudil are eliminated from the vitreous humor either by the anterior or posterior route [22]. It is known that hydrophilic drugs are drained predominantly via the anterior route to the trabecular meshwork. We developed an experimental setup to emulate the situation in the vitreous body and evaluate whether released fasudil disseminates freely within the vitreous humor without significant retention. In this vitreous body diffusion model, fasudil only affects the cells after successful diffusion through the vitreous humor. ECIS measurements were used to monitor the cell response with a time resolution of minutes. The general principle of ECIS is to cultivate adherent cells on gold-film electrodes which allows to measure the changes of the cellular impedance as a function of time. The impedance is determined by the cell type-specific architecture and by the tightness of cell–cell and cell–matrix contacts [65]. It can be measured in a very sensitive and non-invasive manner with high resolution [65]. Our ECIS data clearly demonstrate that fasudil successfully diffuses through the vitreous humor and is thus able to induce changes in cell junctions of TM cells located in a second compartment that is separated from vitreous by a permeable membrane. Despite the diffusion-related time delay through the vitreous humor, the final changes in cell junctions were comparable to those of cells directly treated with fasudil. Our results suggest that the decrease in impedance after treatment with a ROCK inhibitor is attributed to loss of actin stress fibers and focal adhesions. Bischoff et al. treated endothelial cells with the more potent ROCK inhibitor Y-27632 and found a similar impedance time course to those we observed for TM cells directly treated with fasudil [66]. The novelty of our setup is that it includes the diffusion of fasudil through vitreous humor before its cellular effect is monitored by ECIS. Another advantage is that by altering the composition of the gel matrix, the ability of molecules to diffuse through other tissues is assessable in a similar way. Finally, in this study, up to 16 samples were measured simultaneously, and further scaleup to high-throughput formats is easy to realize.

As already mentioned, the remaining fraction of released fasudil will most likely be eliminated via the retina. Besides their neuroprotective effects on retinal ganglion cells [19], ROCK inhibitors are under investigation for the treatment of several vitreoretinal diseases [67]. This could be very beneficial for some glaucoma patients because the prevalence of comorbid retinal disease is higher in patients with primary open-angle glaucoma (15.7%) [68]. For example, intravitreally injected fasudil reduced retinal hypoxia and RPE barrier breakdown in Goto Kakizaki type 2 diabetic rats [69]. Ni et al. investigating the effect of a ROCK inhibitor (Y-27632) on ARPE19 cells, observed increased proliferation and reduced apoptosis of RPE cells [70]. In our study, ARPE19 cells appeared unaffected by treatment with free and released fasudil. Therefore, we assume that intravitreal delivery of a ROCK inhibitor could have a doubly beneficial effect: both on TM and SC cells in the anterior chamber and on retinal cells in the posterior eye.

Regarding the in vivo applicability, a critical question must be raised—are PLGA microspheres a suitable depot for ROCK inhibitors? Subrizi et al. developed a simple equation to calculate the drug dose (D) of an ocular depot formulation necessary for a desired dosing interval (τ) [6]. The equation also takes the drug clearance (CL), bioavailability (F) and the desired steady-state concentration (C_ss,av_) into account:

D = CC_ss,av_ × CL × (τ/F), (4)

This equation allows to predict whether a formulation is suitable as an intraocular delivery device. With regard to our formulation, we used the following parameters and inserted them into the equation: For steady-state concentration C_ss,av_ of fasudil at the target site we assumed 25 µM since it is a widely used concentration in literature and well-tolerated by TM and SC cells [30,31]. The CL of hydrophilic and small molecules from the vitreous is 0.28 mL/h according to literature [6]. For the dosing interval τ, we assumed 30 days based on the mean release duration of fasudil from our microspheres. However, it should be mentioned that longer dosing intervals would be attractive to improve patient compliance. Finally, the bioavailability was considered to be 100% because after intravitreal injection, no tissue barriers must be overcome (F = 1.0). According to this calculation, a total amount of about 1.7 mg fasudil would be required for a 30 day dosing interval. Taking the encapsulation efficacy and the drug loading of the microspheres into account, about 460 mg of microspheres would have to be applied per dosing interval. This polymer amount would correspond to a final intraocular PLGA concentration of 115 mg/mL in humans (volume of human vitreous body: four milliliters [13]). The maximum tolerated concentration of PLGA in the rat vitreous was determined to be about 10 mg/mL [20], thus a total microsphere dose of 460 mg would most likely be too high for intraocular use. According to these considerations, a higher drug content of at least 42.5 µg/mg would be necessary for this application interval. Particularly, when longer dosing intervals are desired, a higher encapsulation efficiency is needed to keep the amount of polymer as low as possible. Because the technical options to increase the encapsulation efficacy of fasudil into PLGA microspheres in an aqueous environment are exhausted, other encapsulation strategies or the encapsulation of other ROCK inhibitors must be considered. The prodrug netarsudil, for example would bring two advantages: first, it is more lipophilic (log*P* = 3.77 [45]) compared to fasudil, which would improve its encapsulation into the lipophilic PLGA matrix. Second, netarsudil is more potent compared to fasudil (netarsudil: K_i_ value _(ROCK2)_ = 1 nM [60]; versus fasudil: K_i_ value _(ROCK2)_ = 330 nM [71]). At least a significant portion of intravitreally released netarsudil will be metabolized to netarsudil–M1 by esterases present in the vitreous humor [72,73,74]. While the lipophilic netarsudil will most likely be eliminated from the vitreous via the posterior route across the retina, the active metabolite netarsudil–M1 (log*P* = 1.19 [45]) will be eliminated via both pathways—the posterior and anterior route [13]. The extent of elimination across the retina depends largely on the localization of the formulation in the vitreous and poses therefore an adjusting screw to control the elimination pathway. In addition, netarsudil–M1 is a more potent ROCK inhibitor compared to netarsudil with a K_i_ value _(ROCK2)_ of 0.2 nM [60], and therefore an even lower concentration will be necessary at the target site to achieve similar effects. We started with fasudil because it is available in sufficient quantities for this type of proof-of-concept study.

## 5. Conclusions

We successfully developed fasudil-loaded PLGA microspheres of different sizes. For one preparation we achieved a controllable sustained release behavior. The biologic activity of released fasudil was successfully demonstrated in several cell-based assays using TM and SC cells as well as fibroblasts. A finding of utmost importance was that the proposed intravitreal application route is feasible, since fasudil is able to overcome the barrier of the vitreous humor and subsequently elicit the desired cellular effect. Because ROCK inhibitors have positive concomitant effects on retinal cells, an intravitreal delivery system could have additional advantages, particularly for glaucoma patients with comorbid retinal diseases. Unfortunately, the encapsulation efficiency of fasudil into the microspheres was quite low, thereby limiting their in vivo applicability. Possibilities to address this issue are to prepare microspheres by altering the lactic to glycolic acid ratio, e.g., with the Resomer^®^ RG 501 H (ratio 45:55). Moreover, spray-drying the microspheres in a nonaqueous system or the manufacture of microspheres using microfluidics could significantly improve the encapsulation efficiency [46]. A completely different approach could be the covalent attachment of fasudil to polyacid poly(L-glutamic acid) based layer-by-layer thin films [75]; or the use of lipid based implants manufactured by compression or extrusion [76,77]. Alternatively, a more lipophilic and potent ROCK inhibitor such as netarsudil could be used, which would be more easily encapsulated into lipophilic polymers such as PLGA and achieve similar efficacy with a lower dose. Our results suggest that intravitreal delivery of a ROCK inhibitor as a dual-purpose drug depot could significantly supplement and improve current glaucoma therapy.

## Figures and Tables

**Table 1:** Parameters for the Preparation of the Microsphere Formulations.

Sample	Method	Volume of Inner Aqueous Phase (mL)	Fasudil (mg)	Volume of DCM (mL)	PLGA (mg)	Stirring Speed (rpm)
W1	W/O/W	1	20	8	200	20,000
W2	W/O/W	0.5	5	3	200	10,000
S	S/O/W	–	20	1	200	7000

DCM: dichloromethane; PLGA: poly (lactic-co-glycolic acid).

**Table 2:** Size, span, encapsulation efficiency (EE), drug loading (DL) and drug content of fasudil-loaded microspheres. Values expressed as mean (± standard deviation) of three independent batches.

Sample	Microsphere Size (µm) ^†^	Size (µm)			Span *	EE (%)	DL (%)	Content (µg/mg Microsphere)
		d (10)	d (50)	d (90)				
W1	3.4 (± 0.6)	1.1 (±0.1)	2.7 (±0.1)	4.5 (±0.6)	1.7 (±0.1)	2.5 (±0.2)	0.2 (±0.0)	2.3 (±0.2)
W2	18.2 (± 2.7)	6.0 (±1.0)	14.7 (±0.7)	32.2 (±4.7)	1.8 (±0.4)	2.3 (±1.0)	0.1 (±0.0)	0.6 (±0.3)
S	66.9 (± 16.0)	30.7 (±1.0)	51.4 (±4.3)	118.6 (±56.4)	1.7 (±1.1)	3.9 (±1.6)	0.4 (±0.2)	3.7 (±1.3)

† mean microsphere size expressed as volume weighted mean diameter D [4.3] * Span = width of the microsphere size distribution: Span = (d90 − d10/d50. d90, d10 and d50 are the microsphere diameters at 90%, 10% and 50% of the cumulative size, respectively.

## Supplementary Materials

The following are available online at https://www.mdpi.com/1999-4923/12/8/706/s1, Figure S1: Fasudil at a concentration of 25 µM is well tolerated by HTM-N cells, TM cells, SC cells and fibroblasts.
