# Supplementary material for: Fasudil Loaded PLGA Microspheres as Potential Intravitreal Depot Formulation for Glaucoma Therapy
Source: Pharmaceutics. 2020 Jul 27;12(8):706. doi: 10.3390/pharmaceutics12080706 (PMC7464914; doi:10.3390/pharmaceutics12080706)
Supplement: Supplementary file 1 [file pharmaceutics-12-00706-s001.pdf]

pharmaceutics

Supplementary Materials: Fasudil Loaded PLGA Microspheres as Potential Intravitreal Depot Formulation for Glaucoma Therapy

Raphael Mietzner, Christian Kade, Franziska Froemel, Diana Pauly, W. Daniel Stamer, Andreas Ohlmann, Joachim Wegener, Rudolf Fuchshofer, Miriam Breunig *

MTT (3-(4,5-dimethylthiazol-2-yl)-2,5-diphenyltetrazolium bromide, a tetrazole)-assay was used to determine the viability of fasudil-treated immortalized trabecular meshwork cells (HTM-N), trabecular meshwork (TM) cells, Schlemm's canal (SC) cells and fibroblasts. MTT is a dye (yellow), which is reduced by cellular enzymes to formazan (blue dye). This transformation is only possible in viable cells; therefore, the amount of formazan is proportional to the number of viable cells [1]. Cells were seeded in a 96-well plate and cultivated for 24 h. Then, cells were washed once with DPBS and the culture medium was replaced by 100 µL of fasudil dilutions ranging from 5 to 1000 µm in culture medium supplemented with 10 or 0.35% FBS and incubated again for 24 h. Two hundred microliters of MTT (625 µg/mL in culture medium without serum) was added and further incubated for six hours at 37 °C. Then, the supernatant was carefully removed, and the MTT crystals were dissolved in 60 µL PBS containing 10% (*w*/*v*) sodium dodecyl sulfate (SDS). The cells were incubated for additional 48 hours in the dark and room temperature. The absorbance of formazan was measured at 570–690 nm using a microplate reader (FLUOstar Omega; BMG Labtech GmbH, Ortenberg, Germany). As positive and negative control served cells treated with DPBS and 0.1% (*w*/*v*) SDS, respectively. The results were calculated as the mean percentage of viability in relation to the positive control. Viabilities below 70% were considered as cytotoxic. (n = 6).

Figure S1 shows the cell viability data of fasudil-treated HTM-N cells, TM cells, SC cells and fibroblasts cells. Data indicate that all analyzed cell types tolerated well a fasudil concentration of 25 µm.

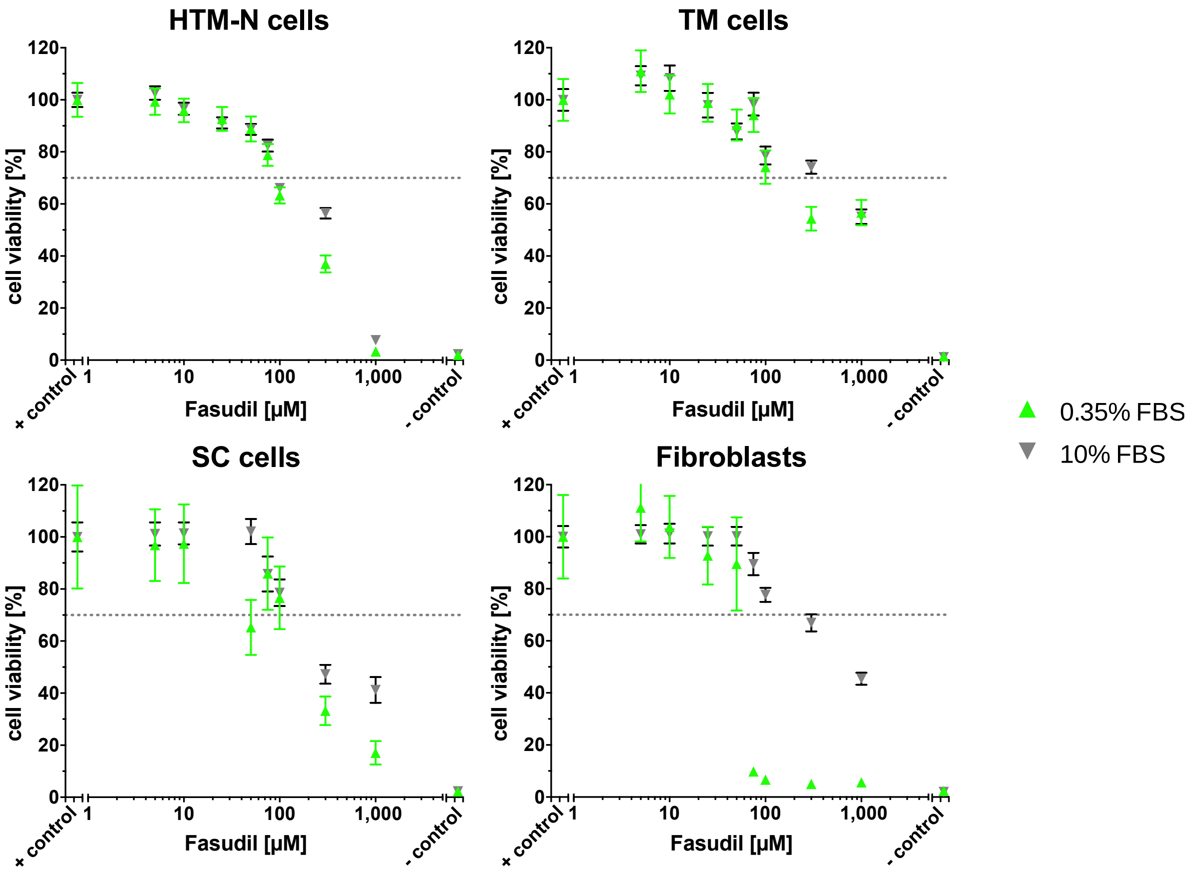

Figure S1. Fasudil at a concentration of 25 µm is well-tolerated by HTM-N cells, trabecular meshwork (TM) cells, Schlemm's canal (SC) cells and fibroblasts. MTT assay data are shown. Cells were incubated for 24 hours with different fasudil concentrations ranging from 5 to 1000 µm, in culture medium supplemented with 0.35 or 10% FBS. All analyzed cell types tolerated well a fasudil concentration of 25 µm. Concentrations below 70% (dashed line) were considered as cytotoxic. Data expressed as mean percentage of viability in relation to the positive control ± standard deviation of the mean (n = 6); + control—positive control (DPBS); - control—negative control (0.1% (w/v) SDS).

References

1. Mosmann, T. Rapid colorimetric assay for cellular growth and survival: Application to proliferation and cytotoxicity assays. *J. Immunol. Methods* **1983**, *65*, 55–63, doi:10.1016/0022-1759(83)90303-4.
